# Healthy Hair Starts With a Healthy Body: Hair Stylists as Lay Health Advisors to Prevent Chronic Kidney Disease

**Published:** 2007-06-15

**Authors:** Sarah L Krein, Mary E Madigan, Linda Smith-Wheelock

**Affiliations:** Department of Veterans Affairs Ann Arbor Healthcare System, Center for Practice Management and Outcomes Research, Ann Arbor, Mich, and University of Michigan Medical School, Department of Internal Medicine, Ann Arbor, Mich; National Kidney Foundation of Michigan, Ann Arbor, Mich; National Kidney Foundation of Michigan, Ann Arbor, Mich

## Abstract

**Background:**

Chronic kidney disease affects one in nine Americans. Diabetes and hypertension account for nearly three quarters of all kidney failure cases. Disproportionate rates of chronic kidney disease, diabetes, and hypertension have been observed among African Americans. More than 70% of all kidney failure cases caused by diabetes and hypertension could have been prevented or delayed with healthy lifestyles and medications.

**Context:**

Approximately 14% of the population living in Michigan is African American. Despite this small proportion, 47% of patients on dialysis and 45% of those on the kidney transplant waiting list are African American. Risk of end-stage kidney failure is 4 times greater among African Americans than among whites.

**Methods:**

The National Kidney Foundation of Michigan developed the Healthy Hair Starts with a Healthy Body (Healthy Hair) campaign to educate African American men and women about their disease risks and to motivate prevention behaviors. The campaign trains African American hair stylists to promote healthy behaviors with their clients through a "health chat" and by providing diabetes and hypertension risk assessment information and incentives.

**Consequences:**

Since 1999, Healthy Hair has trained nearly 700 stylists and reached more than 14,000 clients in eight Michigan cities. Information collected through a client "Chat Form" suggests a number of positive behavioral results.

**Interpretation:**

With nearly 60% of clients indicating that they have taken steps to prevent diabetes, hypertension, and chronic kidney disease or to seek a physician's advice, the Healthy Hair program appears to be effective in the short term in prompting attention to healthy behaviors and increasing risk awareness.

## Background

Chronic kidney disease is serious, common, and costly. One in nine individuals 20 years of age or older have chronic kidney disease (1), which if left untreated usually leads to total kidney failure. Furthermore, an estimated two-thirds of individuals with undiagnosed and untreated diabetes and hypertension, both of which are associated with obesity (2), will develop chronic kidney disease (3). Nationwide, diagnosed and undiagnosed diabetes affect approximately 17 million adults (4), and diagnosed and undiagnosed hypertension, an estimated 65 million (5). In Michigan in 2004, the most recent year for which data are available, diabetes and hypertension accounted for more than 70% of all cases of kidney failure (6). African Americans, who are disproportionately affected by chronic kidney disease, develop hypertension earlier in life than do whites, and their average blood pressure is higher. As a result, the rate of kidney failure is more than 4 times higher for African Americans than for whites (3), and the rate of end-stage kidney disease is 4 times higher (5). With early detection, many, if not most, cases of kidney failure caused by diabetes or hypertension can be prevented or delayed (7). Educating and motivating the population at risk is vitally important. Lifestyle changes to prevent diabetes and hypertension and appropriate treatment of these conditions to reduce the risk of serious complications, such as chronic kidney disease, save both lives and money (8-10).

Numerous public health interventions exist to increase awareness of health issues and to promote behavior change. One particularly promising intervention is the use of lay health advisors (also known as community health workers, peer educators, and lay health workers). One study found that using lay health advisors in combination with a media education campaign was more effective than a media campaign alone in promoting screening for cervical cancer (11). The use of lay health advisors has also been shown to be effective in promoting cancer screening, prevention of sexually transmitted diseases, and smoking cessation among minorities and in immigrant communities (12-16). A study by Flax and Earp (17) suggests that lay health advisors influenced the individuals they counseled because the clients knew the advisors well, felt comfortable talking with them about private issues, and considered them credible sources of information.

This article describes the implementation of a lay health advisor program by the National Kidney Foundation of Michigan (Michigan Kidney Foundation) to increase awareness, promote healthy living, and reduce diabetes, hypertension, and chronic kidney disease in the African American community.

## Context

Approximately 14% of the population living in Michigan is African American. Despite being a relatively small proportion of the population as a whole, African Americans account for 47% of patients on dialysis and 40% of those on the kidney transplant waiting list (6,18). At 354 cases per million people, the annual rate of new cases of chronic kidney disease in Michigan is 5.4% higher than the national average (3). In 2004, diabetes and hypertension, the two leading causes of chronic kidney disease, accounted for more than 70% of all cases of kidney failure in Michigan (3).

Residents in low-income, minority communities have a particularly difficult time engaging in behaviors that can prevent chronic disease. REACH 2010 Surveillance for Health Status in Minority Communities (REACH), which provided data on risk behaviors in 21 communities including Detroit, Michigan, reported that in 2001–2002 minorities were more likely than the general population to be in fair or poor health but not seeing a doctor because of cost (19). REACH also found that the prevalence of eating five or more servings of fruits and vegetables daily and meeting recommendations for moderate or vigorous physical activity was lower for min ority adults than for adults nationally.

The Michigan Surgeon General's report states that as of 2002, approximately 62% of adults in the state were either overweight or obese, and Michigan ranked third highest among the states for obesity (20). Obesity rates were highest for individuals aged 35 to 74 years, African Americans, those with less than a college education, and those with a household income lower than $35,000. African American women have consistently had the highest prevalence of obesity in Michigan.

## Methods

To promote health and reduce diabetes, hypertension, and chronic kidney disease in the African American community, the Michigan Kidney Foundation developed a campaign called Healthy Hair Starts with a Healthy Body (Healthy Hair). The Healthy Hair program began in Detroit in 1999 and relies on hair stylists to educate African American men and women about their disease risks and to help motivate them to adopt behaviors that can lower their risk. This intervention is based on previous work suggesting that individuals, such as hair stylists, who have an established rapport with their clients, can be effective lay educators. Healthy Hair trains African American hair stylists to promote healthy behaviors among their clients through a "health chat," educational materials, and risk assessment information. This project was conducted with approval by the Institutional Review Boards of the University of Michigan Medical School.

The program recruits stylists through direct mailing and personal contact by program staff and current participants. Recruiters appeal to individual interest in improving personal and family health, to community-mindedness, and to the desire to improve business. In a two-part workshop, stylists learn to motivate their clients to make lifestyle changes that can prevent kidney disease, diabetes, and hypertension and to seek medical advice. The workshop emphasizes techniques for motivating clients to improve health behavior and provides instruction on nutrition and exercise.

The workshop follows a curriculum (Appendix A) developed by the Michigan Kidney Foundation in concert with a statewide technical advisory committee, but the content is presented by experts, such as physicians and nutritionists, recruited from each local community. Program coordinators are allowed some latitude on content, and some have included a motivational speaker or a hands-on cooking demonstration to further motivate trainees. The training workshop lasts 4 hours each day and takes place on two consecutive Mondays, traditionally stylists' day off. From there, 12-week campaigns are launched.

The centerpiece of the Healthy Hair salon intervention is the "health chat" offered by stylists (Appendix B). This motivational appeal highlights disease risk factors faced by African Americans and asks clients to take one or more prevention steps: improve diet, increase exercise, stop smoking, or take medication if the client has already been diagnosed with diabetes or hypertension. Stylists also encourage clients to talk with a doctor about their risk for disease, to seek a blood glucose test and urinalysis, and to have their blood pressure measured, as warranted by existing risk factors. Clients complete a risk survey (e.g., the American Diabetes Association's *Take the Test and Know the Score*) as part of the first health chat. American Heart Association videos that reinforce the motivational content are shown at the discretion of the stylist and may not be used in all salons. Educational brochures, posters, and decals reinforce the stylist's message.

At the end of the health chat, the client completes a self-administered survey instrument called a Chat Form, which asks about clients' intentions regarding disease prevention and seeking the advice of a physician. The Chat Form serves as the primary tool for evaluating program outcomes. When the client returns approximately 1 month later, the stylist engages the person in a second health chat, and the client reports successes to date on Part 2 of the Chat Form (Chat 2). Other collected data include disease risk factors, diagnoses, and numbers of clients approached with the campaign message. Six months after the intervention, program staff conduct a telephone survey to identify the potential longer-term impact of the program.

Clients receive a healthy soul-food cookbook at their first visit, and upon completion of Chat 2, they receive a canvas bag containing beauty products and other incentives. Certified diabetes educators telephone clients scoring at high risk for diabetes to encourage them to see a doctor and to reinforce prevention behaviors. Clients at moderate risk are contacted by mail and urged to practice healthy behaviors and to be tested for diabetes.

Stylists receive a $50 stipend for attending the workshop and $4 for each completed Chat Form. They are also offered the opportunity to develop additional leadership skills by serving on a Partners Group, which advises on local campaign issues, including selecting training venues, recruiting volunteer trainers, and accessing local media and resources, and through occasional media and public speaking opportunities. Some veteran stylists go on to assist with campaign training. The estimated cost of the Healthy Hair program, including both direct and indirect costs, is approximately $45 per client served.

## Consequences

Between 1999 and 2005, the Healthy Hair campaign trained nearly 700 stylists and reached more than 14,000 clients in eight Michigan cities: Detroit, Flint, Grand Rapids, Lansing, Muskegon, Pontiac, Saginaw, and Southfield. General characteristics of clients who have participated in the Healthy Hair program are shown in the Table. Both clients and stylists report that the experience is effective and rewarding. Furthermore, self-reported information collected through the Chat Form suggests a number of positive behavioral results. For example, descriptive analyses of data collected on 8148 clients show that of those who indicated at Chat 1 that they planned to eat healthier (n = 7715), 46% reported at Chat 2 that they had increased their fruit and vegetable consumption, were choosing low-fat foods, or were limiting their salt intake. Of those who planned to exercise regularly (n = 6738) at Chat 1, 91% reported at Chat 2 that they continued to exercise at least 3 days per week or more; 32% reported that they had increased the number of days per week in which they exercised. Finally, of those at Chat 1 who indicated that they intended to either eat healthier or exercise regularly, 4545 (56%) reported at least one of the following lifestyle improvements at Chat 2: increased level of exercise, eating more fruits and vegetables, choosing low-fat foods, or limiting salt intake. Besides taking prevention steps, more than 2700 clients reported talking with their doctor during the campaign about their risk for diabetes, hypertension, and kidney disease. Approximately 1750 clients were tested for at least one of these conditions, and 190 were diagnosed with one or a combination of these diseases.

The responses of 60 clients from two cities to a 6-month postintervention survey conducted in spring of 2003 were encouraging both in the number of days of exercise reported and in the number of servings of fruits and vegetables eaten per day. Approximately 60% of survey respondents reported exercising at least 3 to 4 days per week, whereas 41% of the entire cohort reported this behavior at the completion of Chat 2. Eighteen percent reported eating at least five fruits and vegetables per day, a behavior reported by 8% at Chat 2. In one city, 89% of respondents reported limiting salt, and 70% reported limiting fat, rates that surpassed those reported 5 months earlier at Chat 2 and contrary to an expected dip in prevention commitment over time. Moreover, 50% of respondents in each of the two cities reported that campaign information prompted them to change the way they cook for their families, and 70% cited improved shopping habits (e.g., reading labels, selecting lower sodium foods, buying more vegetables). Of note, 75% of those contacted referred to an enduring campaign message that reflected improved knowledge and skills or reinforcement of healthy behaviors. Survey results also helped determine areas for improvement, such as the inclusion of educational brochures for clients to share with their families about healthy eating and cooking.

Many stylists also reported making personal changes in health behavior and said that their businesses have been helped in some way, including improved rapport with clients and a more positive salon atmosphere. In fact, because of the positive experience, several Detroit stylists suggested offering a second phase with different tools and methods. This appeal prompted discussions with statewide technical advisors and funding agencies about appropriate protocols for a follow-up campaign, and a new campaign called Personal Guide to Leading a Healthier Lifestyle, or Phase 2, was generated. Although the original Healthy Hair program was designed primarily to target women, a number of male clients participated in the program. To better address the needs of African American men, we have developed a program with barbers as lay educators. We are now pilot testing this program in several cities.

## Interpretation

Nearly 60% of clients reached by a stylist in the Healthy Hair program reported having taken steps to prevent the targeted diseases or to seek medical advice. Given this outcome, we believe that the intervention is effective in the short term in bringing attention to healthy behaviors and increasing risk awareness. Surveys taken 6 months after the intervention suggested that many clients maintained some behavioral changes and remembered an enduring campaign message. Although these results may be somewhat overstated, considering that they are based on self-reports, they are nonetheless encouraging. Even though the African American urban salon environment appears to be conducive to rendering health advice, characteristics of the industry present challenges. Many stylists work part-time and have other jobs. Because real estate is often inexpensive and plentiful in large cities such as Detroit, salon owners tend to change locations frequently, sometimes moving in the middle of a campaign. Other shops struggle and close before the campaign concludes. Stylists experience some of the same stresses in their lives as do their African American clients, and these have consequences for the program. As a result, 23% of trained stylists fail to complete the campaign.

The advantages of salon-based programming, nonetheless, outweigh the disadvantages. Data gathered in focus groups during campaign development revealed that clients regarded their stylists as trusted advisors, had known them for an average of 8 years, and patronized their shops at least biweekly for an average of 90 minutes at a time. Client comments gathered in 6-month follow-up surveys attest to the comfort level and receptivity many clients have for receiving health information in salons. Considering the many positive aspects of the Healthy Hair campaigns, we have some lessons learned to share after several years of campaign experience:

**Recruitment.** Personal outreach by program staff and multipart marketing that included direct mail, peer-to-peer recruitment, and media blitzes were invaluable in recruiting stylists. The role that financial incentives ($50 stipend for training, $4 per client Chat Form) play in attracting stylists is untested. We know anecdotally that many stylists were drawn to the campaign because of personal experience — either their own or a loved one's — with diabetes, hypertension, or kidney disease.

**Morale.** Mid-campaign luncheons with participating stylists were an effective remedy for lagging commitment and seemed to help in troubleshooting issues that stylists were not otherwise presenting to staff. Occasional gatherings of stylists to foster a shared commitment to improving the African American community through the Healthy Hair campaign also proved helpful.

**Quality.** Client phone surveys conducted 6 months after the campaign revealed a few instances of potential deception by stylists. The financial rewards combined with the complexity of the intervention may have compelled a few stylists to take shortcuts in their approach or, worse, to exploit the campaign. To help mitigate this problem, we stressed quality during training. We also included a modeled Health Chat and one-on-one practice time to emphasize not rushing through the Health Chat. Staff also made salon visits within 1 week of training to observe Health Chat techniques and to correct problems. Monitoring by staff and discussion during training about creating a system for managing client recruitment, Chat Form storage, second-month re-approach, and incentive awards were also useful ways to help maintain quality.

**Support.** Diverse, active, and committed groups of local partners that provided structural support for the campaign were essential. Partners can include voluntary health agencies, hospitals, primary care centers, public health departments, and civic and church groups. Ways in which they can assist include providing volunteer trainers, free training space, educational brochures, cookbooks, videos, incentives, and marketing aid. Some Healthy Hair partners even provided nurses to conduct occasional blood pressure checks in salons. Others promoted the program in their corporate newsletters or featured campaign promotions in their employee cafeteria. We shared campaign results with partners along with information on how to apply local resources to improve behavior outcomes. The identification of local partners with the campaign's mission and success may well ensure long-term sustainability.

The Healthy Hair campaign develops community health resources that will last indefinitely while sustaining existing assets. The campaign turns salons into health information centers where owners and stylists provide knowledge that will stay in the community regardless of the long-term welfare of the campaign. During 2000 through 2005, each participating stylist reached an average of 31 clients, for a total of more than 14,000. Those clients, on average, talked to another three people about what they learned. This ripple effect, coupled with the success in behavior change generated from the Healthy Hair Starts with a Healthy Body campaign, demonstrates the usefulness of reaching at-risk populations through hair salons.

## Figures and Tables

**Table:** Demographic Characteristics of Hair Salon Clients Participating in Healthy Hair Starts with a Healthy Body, Michigan, 2000–2005

Characteristic **a**	Hair Salon Clients, %
**Sex (n = 5,066)**	
Female	73
Male	27
**Age (n = 12,132)**	
18-30	28
31-45	34
46-60	26
>60	11
**Report having health insurance (n = 10,891)**	87

a Denominators vary because of nonresponse and because original data collection forms did not ask about all characteristics.

## Appendices

### Appendix A. Healthy Hair Starts with a Healthy Body

**Training Curriculum Outline**

**Goals**

To develop hair stylists as lay health educators who 1) motivate their clients to adopt behaviors that can prevent kidney disease, diabetes, and hypertension and to seek the care of a physician and 2) increase awareness of the need for organ donation.

**Objectives**

- To educate stylists about kidney disease.
- To motivate stylists to participate in the campaign.
- To equip stylists with skills to successfully motivate clients to make changes.
- To train stylists to use campaign materials and methods appropriately and effectively.
- To ensure that stylists learn the campaign's key messages.

**Day 1 Sessions**

**Understanding Diabetes, Hypertension, and Kidney Failure** (35 minutes)

**Goal:** To equip stylists with basic information about kidneys and strategies for preventing kidney failure, particularly those related to diabetes and hypertension.

**Objectives:** On completion of this session, trainees should

- Be able to describe kidney function and the major risk factors for kidney failure.
- Understand the preventable nature of kidney failure.
- Be familiar with diabetes and hypertension risk factors, warning signs, and strategies for preventing and managing these diseases, including diet, exercise, smoking cessation, and medication compliance.

**Health Status of African Americans (40 minutes)**

**Goal:** To mobilize stylists to work to reduce disease prevalence rates among African Americans.

**Objectives:** On completion of this session, trainees should

- Be able to cite a few key prevalence statistics about kidney failure, diabetes, and hypertension among African Americans.
- Understand some reasons for disproportionate rates of disease, including genetics, behavior choices, health care access and use, and socioeconomic conditions.
- Be motivated to make a difference in disease rates among African Americans.

**Health Chat Demonstration and Practice (30 minutes)**

**Goal:** To prepare stylists to chat confidently and effectively with clients about taking steps toward prevention and seeking the advice of a physician.

**Objectives:** On completion of this session, trainees should

- Be familiar with using the health chat outline.
- Know techniques for overcoming client resistance.
- Know ways to relate campaign goals to their personal story and to articulate what motivates them to take part in the campaign.
- Be focused on using campaign tools effectively.

**Program Tools Overview (45 minutes)**

**Goal:** To familiarize stylists with campaign tools so that they can effectively educate clients and gather campaign data.

**Objectives:** On completion of this session, trainees should

- Be familiar with the campaign brochure, the Chat Form, the brochure *Are You At Risk*, the referral resource card, the 1-month reminder system, and the promotional poster and static decals.
- Understand the importance of program evaluation.
- Be reassured about steps that have been taken to guarantee client confidentiality.
- Know the reasoning behind sensitive questions and be prepared to address issues clients may have with sharing data.

**Day 2 Sessions**

**Nutrition Awareness (45 minutes)**

**Goal:** To increase stylists' awareness of dietary contributors to disease and of strategies for diet improvement.

**Objectives:** On completion of this session, trainees should

- Be able to identify foods that are high in salt and fat and to explain why excess consumption is unhealthy.
- Be familiar with unhealthy cooking techniques and learn strategies for preparing food with less fat and salt.
- Learn how to read a nutrition facts label and how to make healthier choices in eating habits and at the grocery store and fast food restaurants.
- Gain practical tips for getting five servings of fruits and vegetables a day.
- Know about some daily consumption guidelines (e.g., fat, calories, sodium, water) and about healthy serving sizes.
- Be familiar with family-focused nutrition resources such as Michigan State University's county extension and the *Healthy Kids Healthy Weight* brochure.

**Importance of Exercise (45 minutes)**

**Goal:** To equip stylists with practical advice on how to encourage clients with varying degrees of readiness to begin and continue exercising.

**Objectives:** On completion of this session, trainees should

- Understand the basic physiological benefits of exercise.
- Be able to describe some considerations about starting an exercise program.
- Know the minimum daily exercise standards stipulated by the Centers for Disease Control and Prevention.
- Know motivating messages for getting clients to start and maintain an exercise regimen.
- Know about the physiological benefits of smoking cessation and resources for quitting.
- Know some simple exercises that can be done in the salon.
- Be familiar with exercise resources that they can tell their clients about.

**Organ Donation Message (30 minutes)**

**Goal:** To educate stylists about the need for organ donation among African Americans.

**Objectives:** On completion of this session, trainees should

- Know that African Americans have disproportionate rates of dialysis.
- Know that the rate of organ donation is low for African Americans.
- Be acquainted with arguments against organ donation and how to refute them.
- Be familiar with the *Gift of Life* organ donation card.

**Helping Your Clients Cope with Stress (20 minutes)**

**Goal:** To help stylists understand the role stress plays in inhibiting clients from making healthy changes.

**Objectives:** On completion of this session, trainees should

- Understand what stress is, its potential health consequences, and how to recognize it.
- Know how to help clients recognize stress (including the consequences of stress such as hair breakage and nail brittleness) and accept help.
- Be familiar with stress reduction techniques and resources in the community.

### Appendix B. Healthy Hair Starts with a Healthy Body

**Sample Health Chat**

**Introduction**

"I am participating in a women's health project called Healthy Hair Starts with a Healthy Body that is going on in nearly 200 Michigan salons. The goal is to **prevent kidney failure and the two main diseases that cause it, diabetes and high blood pressure**. Our motto is 'We care about more than your hair.' In this salon, my goal is to talk to 25 clients and ask them to consider ways to stay healthy."

Tell your client about your personal motivation to participate. You might say, "I decided to get involved because too many black women are dying of a disease they didn't know they had" or "I am doing this because a close friend died from kidney failure" or

(Write your personal statement here.)

**What I Have Learned**

"Did you know that kidney disease is the seventh leading cause of death for African American women in Michigan? We are at risk because of high rates of high blood pressure and diabetes among African Americans.

"The really bad thing about these diseases is that you can easily have them and not know it. The only way to know if you have high blood pressure is to have your blood pressure checked. The best way to know if you have diabetes is to have a doctor test your blood sugar.

"The great thing is that kidney failure can be prevented 70% of the time by preventing or controlling diabetes and high blood pressure."

**What You Can Do**

"My mission is to get you to think about steps you can take to prevent diabetes and high blood pressure. Even if you already have these diseases, taking steps to prevent complications and to better manage your condition can help you remain healthy longer.

"You can do that in four main ways. [Show client options in the campaign brochure]:

- Eat healthy, well-balanced meals by including five servings of fruits and vegetables per day and limiting salt and fat.
- Exercise regularly. If you don't exercise now, you can begin by walking 10 minutes 3 times a day. If you are already exercising, you should try to get 30 minutes of exercise most days of the week.
- If you're a smoker, stop smoking.
- If you have already been diagnosed with diabetes or high blood pressure, take your medicine."

If you have taken positive steps yourself, you may want to tell your personal story. Something like, "I know it's hard to get started with something like this, and Lord knows I put off making these kinds of changes, but one day I looked at my grandkids and thought, 'You know I've got to do this for them.' Now, I actually like to drink water, and I have more energy because I walk with a girlfriend three times a week."

**What I am Asking You to Do Today**

"For the sake of your health, I am asking you to

- Be open-minded, and read over the materials in the folder I gave you.
- Fill out the risk survey in the brochure *Are You At Risk* that is available here in the salon.
- Choose the prevention steps you will take.
- Write your risk scores and prevention steps on the Chat Form in your folder.
- Come back in a month and fill out the second half of the form. Then I will give you this great bag of goodies. [Show client the bag of incentives.]
- Try out recipes in this cookbook to help get you started. [Give client the campaign cookbook.]
- **If you are at risk for any of these diseases you certainly should see a doctor to be tested.**

"Already over 9500 African American men and women have been reached in Michigan salons with this program and many have learned they have a disease they didn't know they had. Thousands have taken steps to improve their diet and fitness. I just know you will benefit from what I am sharing with you today."

**Second Chat — One Month Later**

"How is it going with the steps you decided to take last time we talked about the Healthy Hair Starts with a Healthy Body program? If you are at risk, were you able to see a doctor? [Praise the client for steps taken. If the client has not started, say you understand how hard it is to make changes but emphasize that doing so can make a definite difference. Inspire the client with a success story from the salon.]

"Here's your Chat Form. Please fill out the yellow section, and I will be happy to give you your goody bag.

"I am so proud of you! It's important to keep up your efforts and not fall back into old habits. Please share the information I've given you with people you care about."
